# Genetic implication of GABA_B_ receptors in the etiology of neurological and psychiatric disorders

**DOI:** 10.3389/fphar.2025.1634128

**Published:** 2025-07-18

**Authors:** Martin Gassmann, Michal Stawarski, Stylianos E. Antonarakis, Bernhard Bettler

**Affiliations:** ^1^ Department of Biomedicine, University of Basel, Basel, Switzerland; ^2^ Medigenome, Swiss Institute of Genomic Medicine, Geneva, Switzerland; ^3^ Department of Genetic Medicine and Development, University of Geneva, Geneva, Switzerland

**Keywords:** *GABBR1*, *GABBR2*, AJAP1, PIANP, neurodevelopmental disorders, epileptic encephalopathy, rett syndrome, autism spectrum disorder

## Abstract

GABA_B_ receptors (GBRs) are G protein-coupled receptors that mediate the actions of the inhibitory neurotransmitter GABA in the central nervous system. Early pharmacological studies with the GBR agonist baclofen and high-affinity antagonists were instrumental in revealing both pre- and postsynaptic functions of GBRs, establishing their critical role in maintaining the excitation-inhibition balance in the brain and highlighting their potential as therapeutic targets. The molecular cloning of GBR subunits enabled the generation of GBR knock-out mouse models, allowing assignment of distinct functions to pharmacologically indistinguishable receptor subtypes and the establishment of causal links between receptor dysfunction and pathological conditions. Advances in high-throughput genomic technologies, particularly whole-exome sequencing, have uncovered hundreds of variants in the genes encoding the GBR subunits, *GABBR1* and *GABBR2*, many of which are linked to neurological and psychiatric disorders. Functional characterization of such variants in recombinant assay systems has revealed both gain-of-function (GOF) and loss-of-function (LOF) mutations, which can now be interpreted in the context of high-resolution structural models of GBR activation. Moreover, proteomic studies have revealed that GBRs form macromolecular complexes with a diverse array of auxiliary proteins that modulate their trafficking, localization, signaling kinetics, and ion channel coupling. Variants in several of these GBR-associated proteins have now also been linked to human disease, with some shown to selectively impair presynaptic GBR functions in relevant mouse models. Here, we review the genetic evidence linking GBR dysfunction to human disease and emphasize the critical role of functional analyses of genetic variants in enhancing diagnostic precision and guiding therapeutic strategies.

## 1 Introduction

**FIGURE 1:**
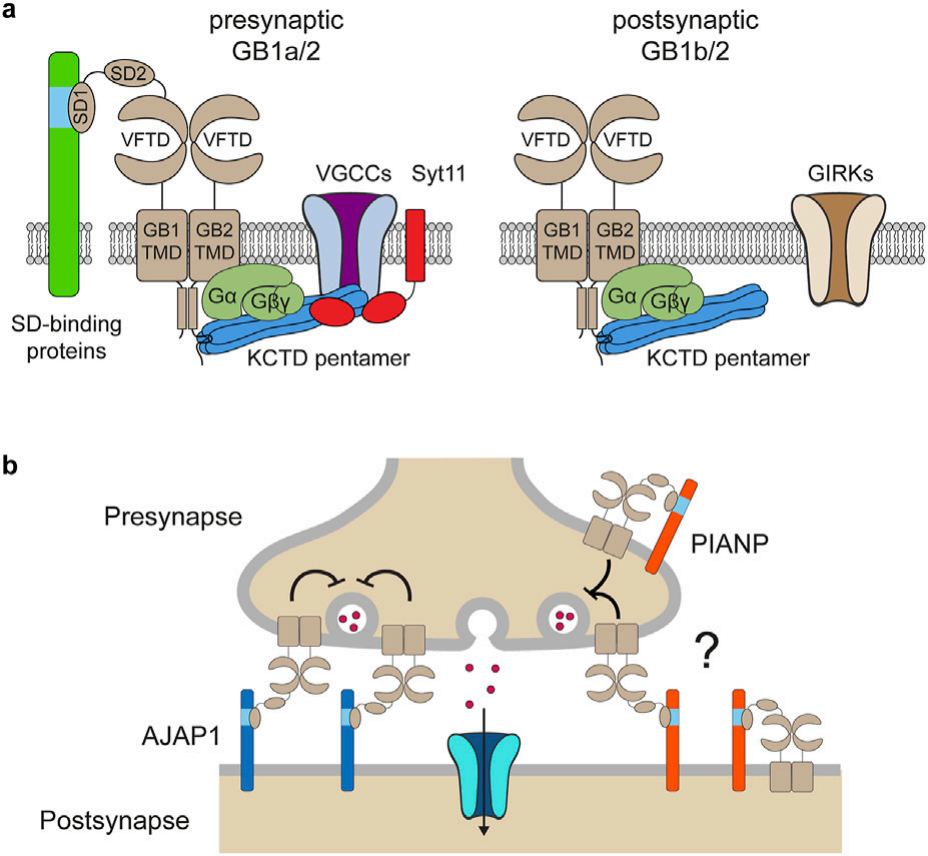
Macromolecular assemblies and functions of pre- and postsynaptic GBRs. **(a)** Presynaptic GBRs are assembled with the GB1a subunit and form a signaling complex with VGCCs to inhibit neurotransmitter release. The assembly of this presynaptic signaling complex is facilitated by KCTD16 and synaptotagmin-11 (Syt11). Additionally, presynaptic receptors interact with sushi domain (SD)–binding proteins via the SD1 of GB1a. Postsynaptic GBRs are assembled with the GB1b subunit and activate G protein-coupled GIRK channels, thereby reducing neuronal excitability. G protein signaling at both pre- and postsynaptic GBRs is modulated by KCTD proteins—auxiliary subunits that bind to the C-terminal domain of GB2 as well as to the G protein βγ subunits. The VFTD of GB1 contains the GABA-binding site, while the TMD of GB2 mediates G protein coupling. **(b)** The dendritically expressed SD-binding protein AJAP1 trans-synaptically recruits GB1a/2 receptors to presynaptic sites. The role of the SD-binding protein PIANP in the context of GBRs remains poorly understood. PIANP is expressed in both axons and dendrites and may interact with presynaptic GB1a/2 receptors either *in cis* or *in trans.*

GBRs were first identified in 1980 by Norman Bowery and colleagues, who used baclofen—a muscle relaxant introduced in 1971 for treating spasticity—to demonstrate the existence of GABA receptors distinct from the ionotropic GABA_A_ receptors (Bowery et al., 1980). GBRs are G protein-coupled receptors that modulate neurotransmission at most synapses in the brain and spinal cord (Gassmann and Bettler, 2012; Pin and Bettler, 2016). They signal through Gi/o-type G proteins to regulate adenylyl cyclases, inwardly rectifying potassium (GIRK or Kir3) channels, and voltage-gated calcium channels (VGCCs). Presynaptic GBRs inhibit the release of both inhibitory and excitatory neurotransmitters by suppressing the activity of VGCCs, while postsynaptic GBRs reduce neuronal excitability by opening GIRK channels, leading to membrane hyperpolarization (Figure 1a). Through these mechanisms, GBRs modulate a broad spectrum of physiological processes, including synaptic plasticity and the regulation of excitation-inhibition balance within neural networks (Gassmann and Bettler, 2012).

Structurally, GBRs are heterodimers composed of GABA_B1_ (GB1) and GABA_B2_ (GB2) subunits, encoded by the *GABBR1* and *GABBR2* genes, respectively. GB1 subunits contain a C-terminal intracellular retention motif that prevents premature surface expression of the receptor. Dimerization with GB2 masks this motif, ensuring that only properly folded and assembled heterodimeric receptor complexes exit the endoplasmic reticulum (Gassmann and Bettler, 2012). Each subunit contains an extracellular venus flytrap domain (VFTD), composed of lobe 1 (LB1) and lobe 2 (LB2), a heptahelical transmembrane domain (TMD), and a C-terminal intracellular domain (Figure 1a) (Frangaj and Fan, 2018; Gassmann and Bettler, 2012; Pin and Bettler, 2016; Shaye et al., 2021). Within the heterodimer, GB1 binds GABA and other orthosteric ligands via its VFTD, while GB2 engages the G protein through its TMD (Mao et al., 2020; Shen et al., 2021). Receptor activation involves conformational changes, including the closure of the GB1 VFTD upon agonist binding, which brings the LB2 lobes of both VFTDs into contact (Frangaj and Fan, 2018; Gassmann and Bettler, 2012; Pin and Bettler, 2016; Shaye et al., 2021). This interaction triggers a rearrangement of transmembrane (TM) helix interfaces from TM3-TM5/TM3-TM5 in the inactive state to TM6/TM6 in the active state, forming a shallow pocket for G protein docking at the base of the GB2 TMD. Competitive antagonists prevent the closure of the GB1 VFTD, while positive allosteric modulators (PAMs) binding at the TM6 interface stabilize the active state of the receptor (Geng et al., 2013; Liu et al., 2021; Mao et al., 2020; Shaye et al., 2021; Shen et al., 2021). Two GB1 isoforms, GB1a and GB1b, are generated from the *GABBR1* gene via alternative promoter usage and splicing (Gassmann and Bettler, 2012). GB1a contains two sushi domains, SD1 and SD2, absent in GB1b (Figure 1a). This structural difference does not affect the orthosteric binding site or alter the signaling properties of GB1a/2 and GB1b/2 receptors, which remain pharmacologically indistinguishable. However, mice lacking GB1a exhibit a loss of presynaptic inhibition of VGCCs, whereas those lacking GB1b show impaired postsynaptic activation of GIRK channels. These findings highlight the critical role of the sushi domains in directing GB1a-containing receptors to presynaptic sites (Gassmann and Bettler, 2012; Vigot et al., 2006).

GBRs form macromolecular complexes through interactions with proteins that influence receptor localization and signaling (Dinamarca et al., 2019; Fruh et al., 2024; Pin and Bettler, 2016; Schwenk et al., 2010; Schwenk et al., 2016). Proteomic studies have identified adherens junction-associated protein 1 (AJAP1), PILR-associated neural protein (PIANP), and potassium channel tetramerization domain-containing proteins—KCTD8, KCTD12, and KCTD16—as being predominantly or exclusively associated with GBRs (Dinamarca et al., 2019; Fruh et al., 2024; Schwenk et al., 2016) (Figure 1a). AJAP1 and PIANP interact with the N-terminal SD1 of the presynaptically expressed GB1a subunit (Dinamarca et al., 2019; Fruh et al., 2024; Schwenk et al., 2016). AJAP1 is selectively expressed in dendrites and recruits GBRs to presynaptic sites through a trans-synaptic mechanism (Dinamarca et al., 2019; Fruh et al., 2024) (Figure 1b). PIANP is expressed in both axons and dendrites, yet its role in the context of GBRs remains poorly understood (Dinamarca et al., 2019; Winkler et al., 2020) (Figure 1b). The KCTD proteins function as auxiliary subunits of GBRs, interacting with the C-terminal domain of GB2 and the Gβγ subunits of the G protein, thereby stabilizing the G protein at the receptor (Fritzius et al., 2024; Turecek et al., 2014) (Figure 1a). This dual interaction with the receptor and the G protein allows KCTD proteins to modulate both the activation and deactivation kinetics of G protein signaling (Fritzius et al., 2024; Schwenk et al., 2010; Turecek et al., 2014). Proteomic analyses have further revealed a broader network of non-exclusive protein interactions with GBRs, including amyloid precursor protein (APP) (Dinamarca et al., 2019; Rem et al., 2023; Rice et al., 2019; Schwenk et al., 2016), synaptotagmin-11 (Syt11) (Trovo et al., 2024), hyperpolarization-activated cyclic nucleotide-gated (HCN) channels (Perez-Garci et al., 2025; Schwenk et al., 2016), VGCCs (Schwenk et al., 2016; Trovo et al., 2024), and transient receptor potential vanilloid 1 (TRPV1) channels (Hanack et al., 2015). APP is required for efficient axonal trafficking of GBRs to presynaptic release sites (Dinamarca et al., 2019), while Syt11 promotes the preassembly of the GBR-KCTD16-VGCC signaling complex prior to its delivery to the plasma membrane (Trovo et al., 2024). Consequently, mice lacking either Syt11 or APP exhibit impaired presynaptic GBR-mediated inhibition of neurotransmitter release (Dinamarca et al., 2019; Trovo et al., 2024). The interaction between HCN channels and GBRs, mediated by KCTD16, facilitates HCN channel activation during postsynaptic hyperpolarization, thereby providing a negative feedback mechanism that curtails the duration of inhibition (Perez-Garci et al., 2025).

Consistent with their essential role in the temporal regulation of neuronal activity and the maintenance of excitation-inhibition balance within neural networks, biochemical and pharmacological studies have now established causal links between variants of uncertain significance (VUS) in the genes for GBR subunits and associated proteins in broad spectrum of neurodevelopmental disorders. These include neurodevelopmental disorder with language delay and variable cognitive abnormalities (NEDLC), neurodevelopmental disorder with poor language and loss of hand skills (NDPLHS), developmental and epileptic encephalopathy 59 (DEE59), intellectual disability (ID), and autism spectrum disorder (ASD). In this review, we explore the role of GBRs in human disease, with particular focus on missense and deletion variants that implicate GBR subunits and key interacting proteins—AJAP1 and PIANP—in disease pathogenesis.

## 2 Expression and autoantibody studies implicating GBRs in disease

Early investigations to explore potential links to disease focused on changes in GBR protein and transcript expression in brain tissue from patients. For example, quantitative autoradiography using [^3^H]-GABA or high-affinity GBR antagonists like [^3^H]-CGP62349, along with immunocytochemistry on hippocampal tissue from patients with temporal lobe epilepsy, supported a reduced GBR density compared to postmortem controls (Munoz et al., 2002; Princivalle et al., 2002; Vlachou, 2022). Altered transcript expression levels and redistribution of GBR subunits have also been observed in the postmortem brains of patients with epilepsy, schizophrenia, autism, bipolar disorder, fragile X syndrome, and Alzheimer’s disease (Fatemi et al., 2009; Fatemi et al., 2017; Iwakiri et al., 2005; Mudge et al., 2008; Sheilabi et al., 2018). Although such expression studies have suggested a role for GBRs in disease, their informative value is limited, as they cannot distinguish whether observed changes in receptor protein or transcript levels reflect adaptive responses to the disease or its treatment, or whether they contribute directly to disease pathogenesis.

Compelling evidence for a direct role of GBRs in the etiology of epilepsy comes from studies showing that autoantibodies targeting GBRs may contribute to autoimmune epilepsy by disrupting receptor expression or interfering with receptor signaling (Lancaster et al., 2010; van Coevorden-Hameete et al., 2019). Notably, autoantibodies against the auxiliary GBR subunit KCTD16 have been detected alongside those targeting the GB1 subunit in patients with encephalitis, further implicating GBRs in the pathogenesis of the disease (van Coevorden-Hameete et al., 2019).

## 3 Pharmacological implications of GBRs in disease

Baclofen (Lioresal^®^), a lipophilic analog of γ-aminobutyric acid (GABA), was initially developed in the 1960s as an antiepileptic agent (Urwyler, 2011). Although it proved ineffective for epilepsy, it was approved in 1971 for the treatment of spasticity associated with conditions such as multiple sclerosis and spinal cord injury. In 1980, baclofen was shown to be a selective agonist of GBRs (Bowery et al., 1980). Baclofen has been explored off-label for various conditions. However, its broader therapeutic application is limited by side effects such as sedation, dizziness, and muscle weakness, as well as by the development of tolerance with prolonged use. Notably, baclofen has been studied extensively for the treatment of alcohol dependence and withdrawal. In 2018, it received formal market authorization in France for the management of alcohol use disorders (Hwa et al., 2014). Gamma-hydroxybutyrate (GHB; Xyrem^®^), a partial agonist at GBRs (Kaupmann et al., 2003), is approved for the treatment of excessive daytime sleepiness and cataplexy in patients with narcolepsy (Roth, 2025). Despite its clinical utility, GHB is classified as a Schedule I controlled substance in the United States outside approved medical use, due to its potent central nervous system depressant effects and high potential for abuse—particularly its involvement in drug-facilitated sexual assault. PAMs of GBRs provide a more selective therapeutic approach than orthosteric agonists, as they enhance the actions of endogenous GABA by increasing the receptor’s affinity and/or efficacy (Urwyler, 2011). PAMs modulate GBRs in a manner that more closely mirrors the receptors’ endogenous temporal and spatial activation patterns, thereby reducing the risk of adverse effects. PAMs of GBRs generally do not produce sedation, hypothermia, or muscle relaxation. Preclinical studies have demonstrated the therapeutic potential of PAMs across a range of conditions, including spasticity, epilepsy, depression, anxiety, pain, and substance use disorders (Bicakci et al., 2022; Cryan and Kaupmann, 2005; Hwa et al., 2014; Jacobson and Cryan, 2008; Kalinichev et al., 2017; Kannampalli et al., 2017; Lopes et al., 2015; Minere et al., 2024; Vlachou, 2022). Although baclofen and PAMs demonstrate that enhancing GBR activity can ameliorate pathological conditions, their therapeutic efficacy alone does not necessarily establish GBR hypofunction as the primary cause of these diseases. Instead, GBR agonists and PAMs are generally expected to be beneficial in disorders characterized by an increased excitation-inhibition ratio within neural networks. Nevertheless, the therapeutic effects of these compounds are often observed in conditions that mirror phenotypes seen in GBR-deficient mice (see 4.1), providing supportive evidence for a causal link between GBR hypofunction and disease pathophysiology. The low-affinity GBR antagonist SGS742 (CGP36742) has demonstrated cognition-enhancing effects in both preclinical and clinical settings (Froestl et al., 2004; Vlachou, 2022). However, broader exploration of GBR antagonists in disease models has been constrained by their proconvulsant liability (Teichgraber et al., 2009; Vergnes et al., 1997), which causally implicates GBR hypofunction in seizure-related hyperexcitability.

## 4 Genetic links between GBRs and disease

### 4.1 GBR-deficient mice

The cloning of GBR cDNAs (Marshall et al., 1999) made it possible to genetically ablate individual receptor subunits in mice, thereby establishing a direct genetic link between GBR dysfunction and disease. Due to the obligate heterodimeric nature of GBRs, knockout of either the GB1 subunit (comprising the GB1a and GB1b isoforms) or the GB2 subunit results in similar synaptic deficits and pathologies (Gassmann and Bettler, 2012; Gassmann et al., 2004; Schuler et al., 2001), including complete loss of both pre- and postsynaptic GBR responses, spontaneous seizures, increased susceptibility to induced seizures, cognitive impairments, hyperactivity, altered circadian activity, and hyperalgesia (Gassmann et al., 2004; Schuler et al., 2001). The occurrence of seizures in GB1 and GB2 knockout mice supports findings from antagonist studies and highlights the key role of GBRs in maintaining the excitation–inhibition balance in the brain through inhibitory signaling. Mice with a heterozygous deletion of the GB1 or GB2 subunits have not been systematically analyzed; however, available data suggest that heterozygous GB1-deficient mice exhibit only mild functional and behavioral deficits (Kaupmann et al., 2003; Schuler et al., 2001). Selective ablation of the GB1a subunit abolishes presynaptic GBR-mediated inhibition of neurotransmitter release, while deletion of the GB1b subunit disrupts postsynaptic inhibition through GIRK channels (Vigot et al., 2006). Notably, only GB1a-deficient but not GB1b-deficient mice exhibit a proconvulsive phenotype (Vigot et al., 2006), highlighting the critical role of presynaptic GBRs in limiting glutamate release and preventing excessive excitation, hypersynchronous network activity, and seizure generation. Similarly, GB1a-deficient mice show pronounced impairments in learning and memory, likely due to disinhibited glutamate release and subsequent saturation of synaptic plasticity mechanisms (Vigot et al., 2006). In comparison, GB1b-deficient mice display milder phenotypes, including hyperactivity, disrupted circadian cycles, spatial memory deficits, and impaired fear conditioning, a form of associative learning (Gassmann and Bettler, 2012). While the therapeutic benefits of baclofen and PAMs largely align with disease phenotypes observed in GBR-deficient mice, the cognition-enhancing effects of the GBR antagonist SGS742 (Froestl et al., 2004) appear at odds with the pronounced learning and memory deficits reported in GBR-deficient mouse models.

### 4.2 Pathogenic *GABBR1* and *GABBR2* variants in humans

Genetic and genomic technologies provide powerful tools for identifying variants in *GABBR1* and *GABBR2* that may predispose individuals to disease or directly contribute its pathogenesis. Given the broad expression of GBRs throughout the central nervous system, and the diverse pathologies observed in GBR-deficient mice, genetic variants that impair receptor function are likely to contribute to disease (Gassmann and Bettler, 2012; Pin and Bettler, 2016). Genome-wide association studies (GWAS Catalog, https://www.ebi.ac.uk/gw intellectual disability as/) have identified single nucleotide polymorphisms and other genetic variants in *GABBR1* and *GABBR2* that are associated with schizophrenia, anxiety and depression/mood disorders, autism spectrum disorder (ASD), post-traumatic stress disorder, alcohol use disorder, insomnia, Alzheimer’s disease, and pain (Table 1). Based on statistical significance and replication across independent cohorts, the strongest genetic associations have been identified for depression and schizophrenia. However, since all GWAS-associated variants in *GABBR1* and *GABBR2* reside in non-coding regions, their impact on GBR function remains unclear. Non-coding variants are thought to influence disease by modulating gene expression or alternative splicing of transcript isoforms. Their regulatory effects are often modest and cell-type specific, which further complicates the functional validation of disease-associated variants (Gallagher and Chen-Plotkin, 2018; Wainberg et al., 2022).

**TABLE 1:** GWAS implicating GBRs in human disease.

Disorder	Number of affected	Gene associated	Most significant SNP	P Value	References
AD	18,892	*GABBR1*	rs148032752 intron variant	2 × 10e-12	Gouveia et al. (2022)
ASD	18,381	*GABBR1*	rs740883 intron variant	1 × 10e-6	Grove et al. (2019)
Depression	224,871	*GABBR1*	rs1235162 intron variant	3 × 10e-16	Dahl et al. (2023)
Depression	113,769	*GABBR1*	rs1233393 intron variant	8 × 10e-13	Howard et al. (2018)
Depression	357,957	*GABBR1*	rs28893517 intron variant	5 × 10e-10	Nagel et al. (2018)
Depression	5919	*GABBR1*	rs28986306 intron variants	2 × 10e-9	Thorp et al. (2021)
Depression	16,301	*GABBR1*	rs926552 3′UTR variant	4 × 10e-8	Cai et al. (2020)
Insomnia	593,724	*GABBR1*	rs28359963 intron variant	4 × 10e-9	Watanabe et al. (2022)
Pain	360,311	*GABBR1* *SUMO2P1*	rs1233380 intergenic variant	2 × 10e-9	Carey et al. (2024)
Schizophrenia	4384	*GABBR1*	rs115070292 intron variant	5 × 10e-10	Yu et al. (2017)
AD	3946	*GABBR2*	rs3824497 intron variant	4 × 10e-6	Sherva et al. (2020)
Alcohol use disorder	8009	*GABBR2* *TBC1D2*	rs10818696 intergenic variant	4 × 10e-6	Benca-Bachman et al. (2023)
Depression	66,200	*GABBR2*	rs80024556 intron variant	2 × 10e-6	Pan et al. (2023)
PTSD	764	*GABBR2*	rs2779551 intron variant	2 × 10e-6	Xie et al. (2013)
Schizophrenia	74,776	*GABBR2*	rs10985811 intron variant	1 × 10e-9	Trubetskoy et al. (2022)
Schizophrenia	96,806	*GABBR2*	rs7869257 intron variant	2 × 10e-8	Dang et al. (2025)
Schizophrenia	37,581	*GABBR2*	rs16914811 intron variant	6 × 10e-7	Goes et al. (2015)
Schizophrenia	47,663	*GABBR2*	rs3824451 intron variant	2 × 10e-7	Ikeda et al. (2019)
Brain size	557	*KCTD8*	rs716890 intron variant	5 × 10e-9	Paus et al. (2012)
ASD	36	*KCTD12* *RN7SL571P*	rs9573902 intergenic variant	9 × 10e-6	Leblond et al. (2019)
Bipolar disorder	1409	*KCTD12* *BTF3P11*	rs2073831 intergenic variant	9 × 10e-6	Lee et al. (2011)
Brain shape	19,670	*KCTD12* *RN7SL571P*	rs4536347 intergenic variant	3 × 10e-8	Naqvi et al. (2021)
Depression	5314	*KCTD12* *RN7SL571P*	rs144999906 intergenic variant	6 × 10e-6	Blokland et al. (2022)
Rumination	1758	*KCTD12* *BTF3P11*	rs674041 intragenic variant	9 × 10e-6	Eszlari et al. (2019)
Alcohol use disorder	272,842	*KCTD16* *RN7SKP246*	rs185177474 intergenic variant	2 × 10e-8	Kranzler et al. (2019)
Insomnia	593,724	*KCTD16* *RN7SKP246*	rs463245 intergenic variant	6 × 10e-11	Watanabe et al. (2022)
Opioid addiction	16,059	*KCTD16* *RN7SKP246*	rs358664 intergenic variant	4 × 10e-5	Gaddis et al. (2022)
Insomnia	593,724	*AJAP1*	rs61765001 5′UTR variant	1 × 10e-8	Watanabe et al. (2022)
Dementia	44,009	*AJAP1* *LINC01646*	rs4654450 intergenic variant	3 × 10e-7	Mega Vascular Cognitive and Dementia (2024)

AD, Alzheimer’s disease; ADHD, attention-deficit/hyperactivity disorder; ASD, autism spectrum disorder; PTSD, post-traumatic stress disorder.

**FIGURE 2:**
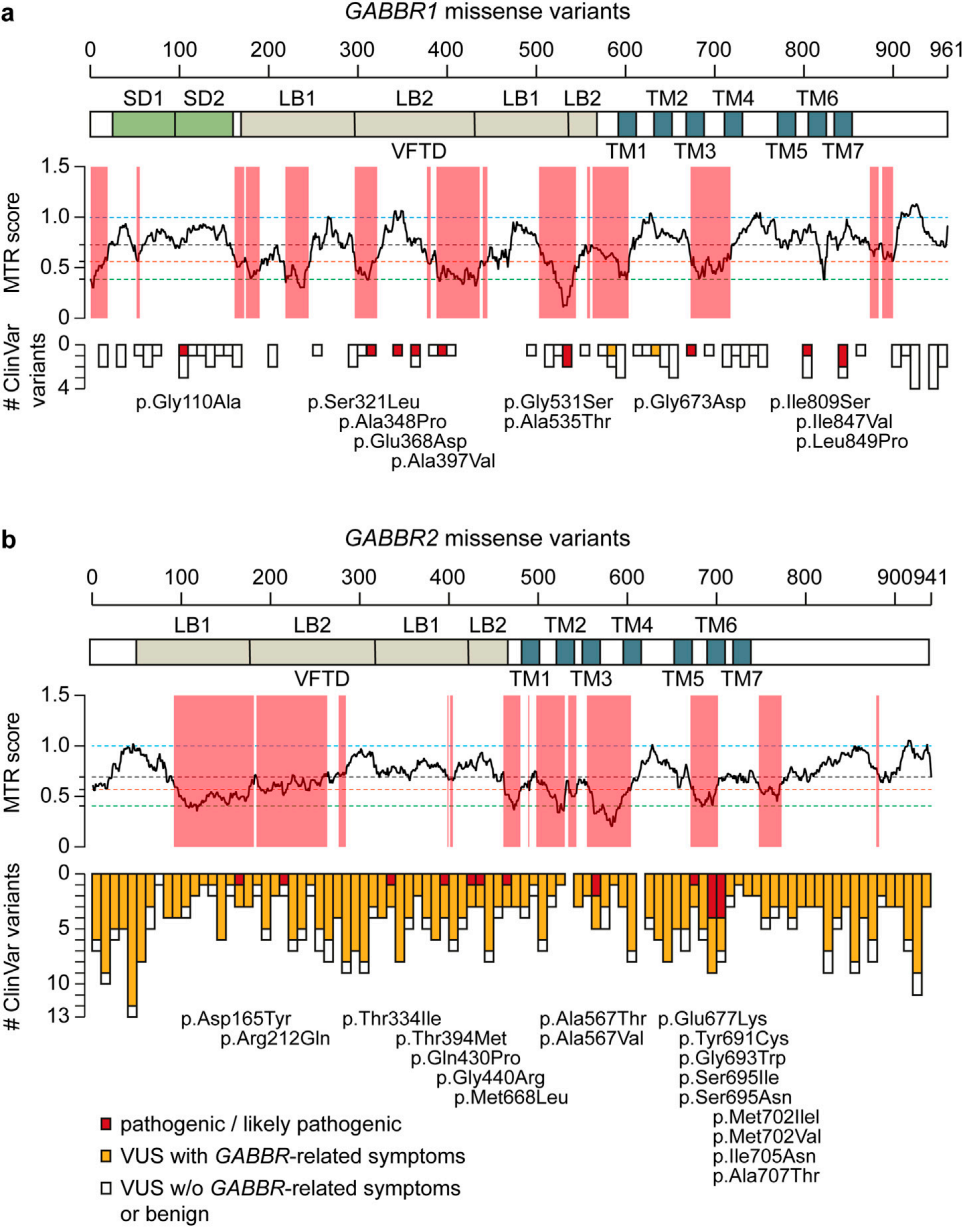
*GABBR1* and *GABBR2* missense variants reported in the ClinVar database and the literature. **(a)** Manhattan plot illustrating the distribution of missense variants in ClinVar along the primary protein sequence of *GABBR1* with a bin size of 10 amino acids. SD1 and SD2 (green), VFTD consisting of LB1 and LB2 (beige) and TM helices (azure) are indicated. Pathogenic and likely pathogenic variants are colored in red and displayed below the plots. VUS associated with phenotypes resembling those caused by functionally validated pathogenic *GABBR1* variants are shown in orange. VUS associated with conditions unlikely to be caused by GBRs are shown in white. The MTR (Traynelis et al., 2017) plotted across the protein-coding sequences is shown. Low MTR scores indicate stronger selection against missense variants. Red-shading indicates protein regions, where the FDR-adjusted binomial exact test, which quantifies MTR deviation from neutrality (MTR = 1), is < 0.1. Horizontal, dashed lines show fifth (green) and 25th (orange) percentiles, median (black), and neutrality (blue). **(b)** MTR ratio and Manhattan plot illustrating the distribution of missense variants in ClinVar along the primary protein sequence of *GABBR2.*

**FIGURE 3:**
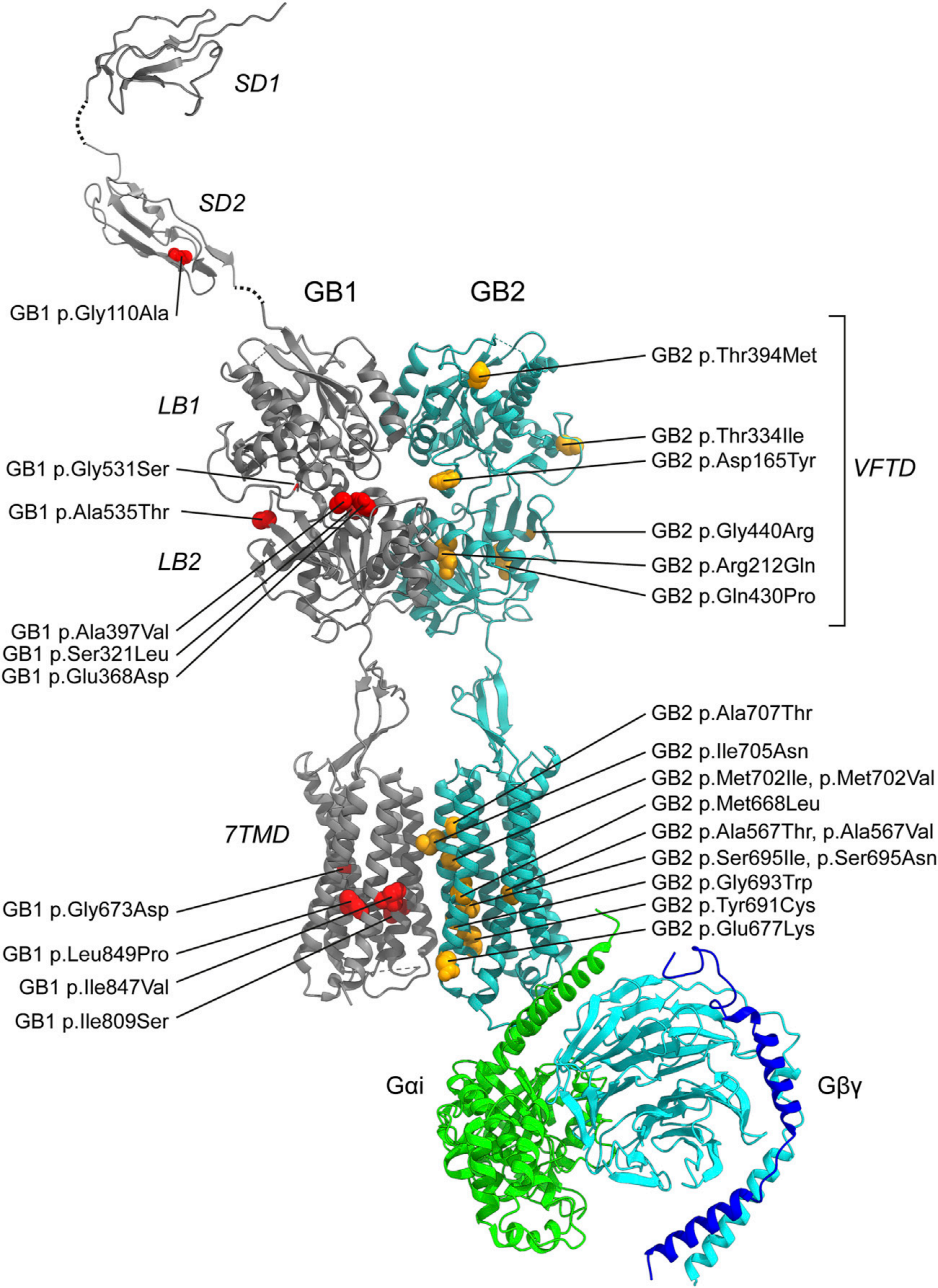
Pathogenic GBR missense variants. The model integrates published structures of the baclofen-bound GBR–Gα_i_ protein complex (PDB: 7EB2 (Shen et al., 2021)), Sushi Domain 1 (SD1; PDB: 6HKC (Rice et al., 2019)), and Sushi Domain 2 (SD2; PDB: 1SRZ (Blein et al., 2004)). GB1 and GB2 subunits are shown in dark grey and sea blue, respectively. The G protein components are colored as follows: Gα_i_, bright green; Gβ, cyan; Gγ, dark blue. Structural domains of GB1 and GB2 (SD1, SD2, LB1, LB2, VFTD, 7TMD) are labeled in italics. Pathogenic variants are indicated in red for GB1 and yellow for GB2. Notably, GB1 p.Ser321Leu and GB1 p.Glu368Asp, both located near the orthosteric binding site within the VFTD of GB1, decrease GABA potency. In contrast, GB1 p.Gly531Ser, situated in a hinge region outside the orthosteric site, and GB2 p.Arg212Gln within the VFTD of GB2, induce constitutive activity. Additionally, several variants located in the 7TM domains of GB1 and GB2 also increase constitutive activity, including GB1 p.Ile809Ser (TM6), GB1 p.Ile847Val (TM7), GB2 p.Ala567Thr (TM3), GB2 p.Ser695Ile (TM6), GB2 p.Met702Val (TM6), GB2 p.Ile705Asn (TM6), and GB2 p.Ala707Thr (TM6). See Tables 2,3 for detailed information on the specific locations of other variants.

In contrast to non-coding GWAS variants, missense variants identified through whole-exome sequencing (WES) in affected individuals offer a more direct and potentially causal link to disease. *GABBR1* and *GABBR2* are classified as haploinsufficient genes, as indicated by their LOF intolerance (pLI) scores of 1 in the gnomAD database (https://gnomad.broadinstitute.org/), indicating strong selective pressure against protein-truncating variants. In contrast, mouse models with heterozygous deletion of *Gabbr1* exhibit only mild functional or behavioral deficits (Kaupmann et al., 2003; Schuler et al., 2001), suggesting species-specific differences in dosage sensitivity or compensatory mechanisms. Both genes also exhibit significant constraint against missense variation, with missense Z-scores of 5.54 (*GABBR1*) and 4.11 (*GABBR2*) in gnomAD, suggesting that protein-altering mutations are generally not well tolerated and are more likely to be deleterious and potentially disease-causing. ClinVar (https://www.ncbi.nlm.nih.gov/clinvar/), a database documenting human genetic variants and their clinical significance, reports 80 missense variants in *GABBR1* and 433 in *GABBR2* (Figure 2). Among these, seven monoallelic *de novo* variants in *GABBR1* and fourteen in *GABBR2* are classified as pathogenic or likely pathogenic. Additional variants with strong evidence of pathogenicity have been reported in the literature but have not yet been included into ClinVar. These variants are listed in Table 2 (*GABBR1*) and Table 3 (*GABBR2*), and have been mapped onto the structural model of GBRs (Figure 3). The missense tolerance ratio (MTR) provides a codon-level measure of selective constraint derived from human population sequencing data (Traynelis et al., 2017). Many, though not all, pathogenic variants in *GABBR1* and *GABBR2* cluster in regions with low MTR scores, consistent with strong purifying selection against amino acid substitutions in these regions (Figure 2). Due to limited functional validation and incomplete clinical annotation, most missense variants in these genes are currently classified as VUS (Nykamp et al., 2017). Nonetheless, several of these VUS have been identified in individuals with phenotypes consistent with GBR-related disorders (Figure 2). Notably, many of these VUS are located in low-MTR regions, particularly within *GABBR2*, supporting that they may be pathogenic and warrant further investigation.

**TABLE 2:** *GABBR1* missense variants.

Protein	Receptor	Condition	gnomAD (v.4.1.0)	CADD	REVEL	AlphaMissense	Functional validation	References
p.Gly110Ala	SD2	NEDLC	absent	27.7	0.735	0.7648	no	ClinVar
p.Ser321Leu	VFTD	NEDLC, epilepsy	6.20e-7	33	0.675	0.8519	yes	
p.Glu368Asp	VFTD	NEDLC, epilepsy	absent	24.1	0.605	0.9925	yes	ClinVar (Cediel et al., 2022)
p.Ala397Val	VFTD	NEDLC, ADHD	absent	32	0.668	0.8618	yes	ClinVar (Cediel et al., 2022)
p.Gly531Ser	VFTD	NEDLC, ASD	absent	32	0.874	0.9945	yes	DECIPHER
p.Ala535Thr	VFTD	NEDLC	absent	29.5	0.586	0.9261	yes	ClinVar (Cediel et al., 2022)
p.Gly673Asp	TM3	NEDLC, ASD, ADHD	absent	31	0.951	0.9993	yes	ClinVar (Cediel et al., 2022)
p.Ile809Ser	TM6	NEDLC, ASD, ADHD, epilepsy	absent	32	0.897	0.996	yes	
p.Ile847Val	TM7	NEDLC, ASD	absent	23.9	0.507	0.4329	yes	
p.Leu849Pro	TM7	NEDLC	absent	29.9	0.964	0.999	no	ClinVar

Score thresholds: CADD (range 0–99) benign ≤22.7, deleterious ≥25.3; REVEL (range 0–1) benign ≤0.29, deleterious ≥0.644; AlphaMissense (range 0–1) benign <0.34, deleterious >0.654. ASD, autism spectrum disorder; ADHD, attention-deficit/hyperactivity disorder; NEDLC, neurodevelopmental disorder with language delay and variable cognitive abnormalities (OMIM #620502).

**TABLE 3:** *GABBR2* missense variants.

Protein	Receptor	Condition	gnomAD (v.4.1.0)	CADD	REVEL	Alpha Missense	Functional validation	References
p.Asp165Tyr	VFTD	NDPLHS	absent	30	0.495	0.9335	yes	ClinVar
p.Arg212Gln	VFTD	NDPLHS, ASD	absent	24.5	0.486	0.6491	yes	ClinVar (Bielopolski et al., 2023)
p.Thr334Ile	VFTD	epileptic encephalopathy	absent	33	0.622	0.9453	no	
p.Thr394Met	VFTD	global developmental delay, epileptic encephalopathy	3.098e-5	25.4	0.307	0.096	no	ClinVar DECIPHER
p.Gln430Pro	VFTD	NDPLHS, ADHD, ASD	absent	28	0.537	0.998	yes	
p.Gly440Arg	VFTD	NDPLHS, epilepsy	absent	29.8	0.738	0.9987	no	Samanta and Zarate (2019)
p.Ala567Thr	TM3	NDPLHS, epileptic encephalopathy	absent	28	0.749	0.8207	yes	ClinVar DECIPHER (Carneiro et al., 2018; Lopes et al., 2016; Takata et al., 2018; Yoo et al., 2017; Vuillaume et al., 2018)
p.Ala567Val	TM3	epileptic encephalopathy	absent	32	0.833	0.9385	no	ClinVar
p.Met668Leu	TM5	Infantile-onset epilepsy	absent	22	0.597	0.3872	no	Kim et al. (2021)
p.Glu677Lys	TM5-TM6 cytoplasmic loop	DEE59	absent	32	0.808	0.9974	no	ClinVar
p.Tyr691Cys	TM5-TM6 cytoplasmic loop	DEE59	absent	32	0.931	0.9603	no	ClinVar
p.Gly693Trp	TM6	DEE59	absent	33	0.931	0.9995	yes	ClinVar (Bertoli-Avella et al., 2021; D'Onofrio et al., 2022; Hamdan et al., 2017; Minere et al., 2024)
p.Ser695Ile	TM6	DEE59	absent	32	0.97	0.9968	yes	ClinVar; EuroEPINO MICS 2014 (Hamdan et al., 2017; Minere et al., 2024; Yoo et al., 2017; Vuillaume et al., 2018)
p.Ser695Asn	TM6	DEE59, IESS, NDPLHS	absent	31	0.851	0.9931	no	ClinVar (Nagarajan et al., 2023)
p.Met702Ile	TM6	ID	absent	29.7	0.792	0.9951	no	ClinVar
p.Met702Val	TM6	NDPLHS	absent	24.2	0.77	0.826	yes	
p.Ile705Asn	TM6	DEE59	absent	33	0.909	0.9953	yes	ClinVar; EuroEPINO MICS 2014 (Minere et al., 2024; Yoo et al., 2017; Vuillaume et al., 2018)
p.Ala707Thr	TM6	DEE59 NDPLHS	absent	26.3	0.772	0.8568	yes	ClinVar (Marinakis et al., 2021; Vuillaume et al., 2018)

Score thresholds: CADD (range 0–99) benign ≤22.7, deleterious ≥25.3; REVEL (range 0–1) benign ≤0.29, deleterious ≥0.644; AlphaMissense (range 0–1) benign <0.34, deleterious >0.654. ASD, autism spectrum disorder; ADHD, attention-deficit/hyperactivity disorder; ID, intellectual disability; IESS, infantile epileptic spasms syndrome; NDPLHS, neurodevelopmental disorder with poor language and loss of hand skills (OMIM #617903); DEE59, developmental and epileptic encephalopathy 59 (OMIM #617904).

**FIGURE 4:**
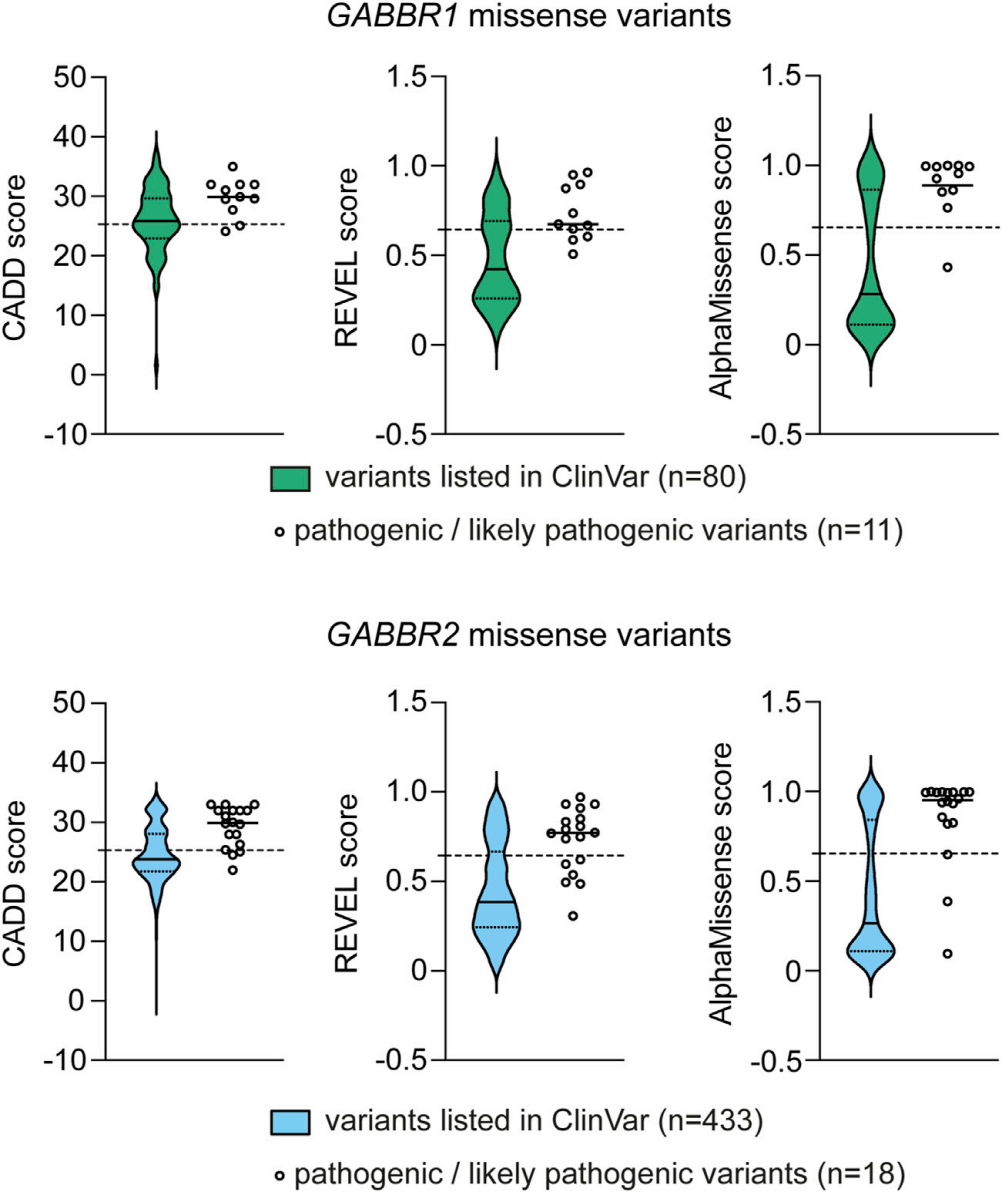
Computational pathogenicity prediction scores for missense variants listed in ClinVar (source: dbNSFP v.5.1). Variants were assessed using the CADD, REVEL and AlphaMissense prediction tools. Data are shown as violin plots, with the median (solid black line) and the first and third quartiles (dotted lines) indicated. For comparison, the scores of pathogenic/likely pathogenic variants from Tables 2,3 (*GABBR1* and *GABBR2*) are shown on the right, with their median values marked. The scores for each of these variants are provided in Tables 2,3. Dashed horizontal lines denote the deleteriousness thresholds specific to each prediction tool, above which variants are predicted to affect protein function and are thus considered potentially pathogenic.

Several algorithms have been developed to predict the pathogenicity of single nucleotide variants (MacArthur et al., 2014). These algorithms are used in conjunction with variant frequency data from case cohorts and reference population databases, such as gnomAD, BRAVO, and Regeneron (Karczewski et al., 2020). To further assess the potential pathogenicity of *GABBR1* and *GABBR2* VUS associated with GBR-related disorders, we used three *in silico* prediction tools: REVEL (Ioannidis et al., 2016), CADD (Kircher et al., 2014), and AlphaMissense (Cheng et al., 2023). Scores for REVEL, CADD and AlphaMissense were obtained from the dbNSFP database (https://www.dbnsfp.org/) and compared to those of known pathogenic variants (Figure 4). The results show that all three tools reliably classify the majority of known pathogenic variants as deleterious. Notably, many *GABBR1* and *GABBR2* VUS linked to GBR-related disorders also received high pathogenicity scores, suggesting they may be disease-causing.

Pathogenic variants in *GABBR1* are commonly associated with a clinical phenotype that includes neurodevelopmental delay and/or epilepsy (Cediel et al., 2022). Affected individuals typically present with early motor delays, speech and language impairments, ID, learning difficulties, and/or behavioral abnormalities. This phenotypically heterogeneous neurological disorder, caused by monoallelic *de novo* missense *GABBR1*, is designated as NEDLC. Pathogenic variants in *GABBR2* were initially identified in individuals with DEE59 (Euro et al., 2014) or with clinical features resembling atypical Rett syndrome (Lopes et al., 2016; Vuillaume et al., 2018; Yoo et al., 2017). Individuals with the latter presentation are now more accurately diagnosed with NDPLHS. This condition is characterized by developmental stagnation or regression in early childhood, typically manifesting as loss of purposeful hand movements, impaired or lost language abilities, and ID. Since these initial reports, additional pathogenic *de novo* mutations in *GABBR2* have been discovered in cohorts of individuals with ID (Carneiro et al., 2018; Deciphering Developmental Disorders, 2017), ASD (Al-Sarraj et al., 2021; Takata et al., 2018), and drug-resistant epilepsy (Kim et al., 2021; Rochtus et al., 2020). Notably, the recurrent *de novo* missense variant *GABBR2* p.Ala567Thr, which affects a highly conserved residue within the third transmembrane helix (TM3), has been identified in more than 10 unrelated individuals presenting with NDPLHS.

While computational predictions are valuable for assessing the potential pathogenicity of variants, functional studies are essential to determine their impact on protein function, including whether they cause GOF or LOF effects and to what extent these alterations influence receptor activity. Furthermore, functional studies help elucidate molecular disease mechanisms—an essential step toward accurate diagnosis and the development of targeted therapies. Cell-based assay systems that enable direct and selective measurement of GBR activity have proven to be both cost-effective and highly informative for functionally characterizing missense variants in *GABBR1* and *GABBR2* (Bielopolski et al., 2023; Cediel et al., 2022; Vuillaume et al., 2018). These analyses have revealed a number of functional alterations in pathogenic variants, which may also occur in combination: (i) reduced or absent surface expression, leading to decreased or abolished GABA efficacy at the receptor; (ii) a significant reduction in the potency of GABA at the receptor; and (iii) increased constitutive activity. A reduction in surface expression is observed for several pathogenic variants in *GABBR1* and *GABBR2*, which can be located either in the extracellular VFTD or within the TMD. Notably, *GABBR1* p.Gly673Asp in TM3 and *GABBR2* p.Gln430Pro in the VFTD fail to reach the cell surface, rendering the receptors completely inactive. A decrease in GABA potency is observed at *GABBR1* p.Glu368Asp and p.Ser321Leu, both situated in the VFTD near the orthosteric binding site. These variants likely decrease the receptor’s affinity for GABA. Increased constitutive activity is predominantly associated with variants located in the TMDs of GB1 and GB2. Specifically, *GABBR1* p.Ile809Ser and p.Ile847Val, along with *GABBR2* p.Ala567Thr, p.Ser695Ile, p.Met702Val, p.Ile705Asn, and p.Ala707Thr, all exhibit enhanced constitutive activity to varying degrees. Constitutive activity in these variants is reversed by the competitive GBR antagonist CGP54626, except for p.Ser695Ile, which is fully active in the absence of GABA (Vuillaume et al., 2018). Structural mapping of these variants onto available GBR models reveals their localization along the TMDs (Figure 3). Structural data suggest that amino acid substitutions within the TMDs can stabilize the active state of GB2, thereby enabling G protein activation even in the absence of GABA binding (Liu et al., 2021; Shaye et al., 2021). Increased constitutive activity is also observed with variants in the VFTDs. Molecular dynamics simulations suggest that *GABBR1* p.Gly531Ser, located in a hinge region outside the orthosteric binding site, and *GABBR2* p.Arg212Gln, situated in the VFTD, both induce local conformational changes that stabilize the active state of the receptor. This aligns with the allosteric activation mechanism of GBRs, in which agonist binding to the VFTD of GB1 induces conformational changes transmitted to the TMD of GB2, ultimately activating the G protein (Shaye et al., 2021). Notably, due to their elevated baseline activity in the absence of GABA, all constitutively active variants exhibit a corresponding reduction in GABA efficacy. The *GABBR1* variant p.Gly110Ala, situated in SD2, has not yet been functionally characterized, but may selectively impair the function of presynaptic GBRs. Overall, functional studies have revealed both LOF and GOF variants in *GABBR1* and *GABBR2*.

The *in vivo* effects of constitutively active variants are likely to be complex and context-dependent. Under conditions of low ambient GABA, constitutive activity and increased GABA potency may enhance GBR signaling, producing a GOF effect. In contrast, during periods of elevated synaptic GABA concentrations, reduced GABA efficacy could lead to a net LOF. While inverse agonists can suppress constitutive receptor activity, they risk further dampening GABA-mediated signaling during synaptic transmission, potentially exacerbating functional deficits. Functional studies in transfected neurons suggest that certain constitutively active *GABBR2* variants disrupt receptor trafficking to the neuronal surface, resulting in reduced signaling efficacy and contributing to presynaptic hyperexcitability (Minere et al., 2024). Notably, this synaptic phenotype was reversed by pharmacological enhancement of GBR signaling using a PAM. Moreover, variants such as *GABBR2* p.Ser695Ile, which exhibit high constitutive activity, may trigger adaptive cellular mechanisms that ultimately downregulate receptor function. These observations underscore the challenge of selecting an optimal therapeutic strategy based solely on *in vitro* data, emphasizing the need for a deeper understanding of variant-specific effects in a physiological context. The advent of CRISPR/Cas genome editing has made it relatively rapid and cost-effective to generate mouse models carrying specific variants inserted into the endogenous gene locus. Such models closely replicate the human condition by maintaining physiological expression levels within the native neuronal environment—a critical factor when studying monoallelic variants. These models offer a powerful platform for detailed investigations of synaptic and network function through both *in vitro* and *in vivo* electrophysiology. In parallel, they enable comprehensive biochemical profiling of the receptor and its signaling partners, facilitating the identification of adaptive or compensatory mechanisms that may emerge in response to altered receptor function.

Interestingly, both LOF and GOF variants can give rise to overlapping clinical phenotypes (Table 4). As noted above, GOF effects driven by constitutive receptor activity are accompanied by a reduced responsiveness to synaptic GABA, effectively resulting in a concomitant LOF. In addition, both types of variants may disrupt homeostatic mechanisms critical for maintaining neural network stability and the balance between excitation and inhibition (Vertkin et al., 2015). Such disruption likely contributes to the etiology of neurological and psychiatric disorders, including epilepsy, ID, and ASD (Issa et al., 2023; Wondolowski and Dickman, 2013).

**TABLE 4:** GOF and LOF variants in *GABBR1* and *GABBR2*.

Protein	Gene	Effect	Pharmacology	Surface expression	Condition
p.Gly531Ser	*GABBR1*	GOF	full constitutive activity	reduced	NEDLC, ASD
p.Ile809Ser	*GABBR1*	GOF	partial constitutive activity, increased potency	normal	NEDLC, ASD, ADHD, epilepsy
p.Ile847Val	*GABBR1*	GOF	partial constitutive activity, increased potency	normal	NEDLC, ASD
p.Asp165Tyr	*GABBR2*	GOF	partial constitutive activity, increased potency	reduced	NDPLHS
p.Arg212Gln	*GABBR2*	GOF	partial constitutive activity, increased potency	reduced	NDPLHS, ASD
p.Ala567Thr	*GABBR2*	GOF	partial constitutive activity	normal	NDPLHS, epileptic encephalopathy
p.Ser695Ile	*GABBR2*	GOF	full constitutive activity	normal	DEE59
p.Met702Val	*GABBR2*	GOF	partial constitutive activity, increased potency	normal	NDPLHS
p.Ile705Asn	*GABBR2*	GOF	partial constitutive activity	normal	DEE59
p.Ala707Thr	*GABBR2*	GOF	partial constitutive activity	normal	DEE59, NDPLHS
p.Ser321Leu	*GABBR1*	LOF	reduced potency	normal	NEDLC, epilepsy
p.Glu368Asp	*GABBR1*	LOF	reduced potency, reduced efficacy	reduced	NEDLC, epilepsy
p.Ala397Val	*GABBR1*	LOF	reduced efficacy	normal	NEDLC, ADHD
p.Ala535Thr	*GABBR1*	LOF	reduced efficacy	normal	NEDLC
p.Gly673Asp	*GABBR1*	LOF	no response	absent	NEDLC, ASD, ADHD
p.Gln430Pro	*GABBR2*	LOF	no response	absent	NDPLHS, ADHD, ASD

Main pharmacological effects and associated conditions of gain-of-function (GOF) and loss-off-function (LOF) variants in *GABBR1* and *GABBR2*. ADHD, attention-deficit/hyperactivity disorder; ASD, autism spectrum disorder; DEE59, developmental and epileptic encephalopathy 59 (OMIM #617904); NDPLHS, neurodevelopmental disorder with poor language and loss of hand skills (OMIM #617903); NEDLC, neurodevelopmental disorder with language delay and variable cognitive abnormalities (OMIM #620502).

### 4.3 *AJAP1* variants

Proteomic analyses of brain tissue have identified AJAP1 as a primary interaction partner of GBRs (Schwenk et al., 2016). AJAP1 is a single-pass transmembrane protein broadly expressed in neurons, with its extracellular domain binding to SD1 of GB1a (Figure 1a) (Dinamarca et al., 2019). Through this interaction, AJAP1 trans-synaptically recruits GB1a-containing GBRs to presynaptic sites, thereby influencing their synaptic localization and function (Figure 1b) (Fruh et al., 2024).

Genetic variants in genes encoding GBR-associated proteins, such as AJAP1, may contribute to diseases resulting from GBR dysfunction. GWAS studies have implicated non-coding *AJAP1* variants in insomnia and dementia (Table 1). WES and chromosomal microarray analysis have identified individuals carrying either the *AJAP1* missense variant p.Trp183Cys, the frameshift variant p.I271Ffs*24, the splice-site variant c.917 + 1G>C, or a complete deletion (Table 5) (Fruh et al., 2024). These individuals predominantly present with global developmental delay, ID, hypotonia, and/or epileptic seizures. These clinical features closely resemble those reported in individuals with LOF variants in *GABBR1* (Table 2) (Cediel et al., 2022), indicating that impaired GBR function may contribute to the underlying pathogenesis. The *de novo* AJAP1 variant p.Trp183Cys replaces a critical tryptophan at position 183 that is essential for SD1 binding. The *de novo* p.I271Ffs*24 frameshift variant may trigger nonsense-mediated mRNA decay. However, any transcript escaping decay is expected to produce a truncated protein lacking the transmembrane and intracellular domains, while retaining the SD1 binding site. A paternally inherited complete *AJAP1* deletion results in LOF. The splice-site variant c.917 + 1G>C is predicted to disrupt normal splicing, potentially leading to nonsense-mediated decay, exon skipping, activation of a cryptic splice site, or intron retention.

**TABLE 5:** *AJAP1* and *PIANP* missense and deletion variants.

Gene	Protein/Variant	Conditions	gnomAD (v.4.1.0)	CADD	REVEL	Alpha Missense	Functional validation	References
*PIANP*	p.Arg172Pro heterozygous	musculoskeletal and nervous system abnormalities	absent	24.5	0.198	0.1709	no	DECIPHER
*PIANP*	p.Arg114* homozygous	global developmental delay, bilateral cryptorchidism, hypotonia	6.20e-7	38.0			no	Anazi et al. (2017)
*AJAP1*	p.Trp183Cys heterozygous	epilepsy	absent	29.3	0.759	0.9982	yes	Fruh et al. (2024)
*AJAP1*	p.Pro242Ser nonmaternal (father not available)	epilepsy, global developmental delay, motor delay, nonverbal, hypertonia, ID	1.25e-6	21.9	0.042	0.0875	yes, benign	Fruh et al. (2024)
*AJAP1*	p.Ile271Phefs*24 heterozygous	epilepsy, global developmental delay, motor delay, nonverbal, ASD, tourette syndrome hypotonia	absent				yes	Fruh et al. (2024)
*AJAP1*	*AJAP1* deletion chr1:4,505,547-5,384,043 (hg38) paternal (mosaic)	speech delay, epilepsy, ID, hypotonia					no	Fruh et al. (2024)
*AJAP1*	c.917 + 1G>C NM_018836.4 heterozygous	speech delay, ID	absent	35			no	Fruh et al. (2024)

Score thresholds: CADD (range 0–99) benign ≤22.7, deleterious ≥25.3; REVEL (range 0–1) benign ≤0.29, deleterious ≥0.644; AlphaMissense (range 0–1) benign <0.34, deleterious >0.654. ASD, autism spectrum disorder; ID, intellectual disability.

**FIGURE 5:**
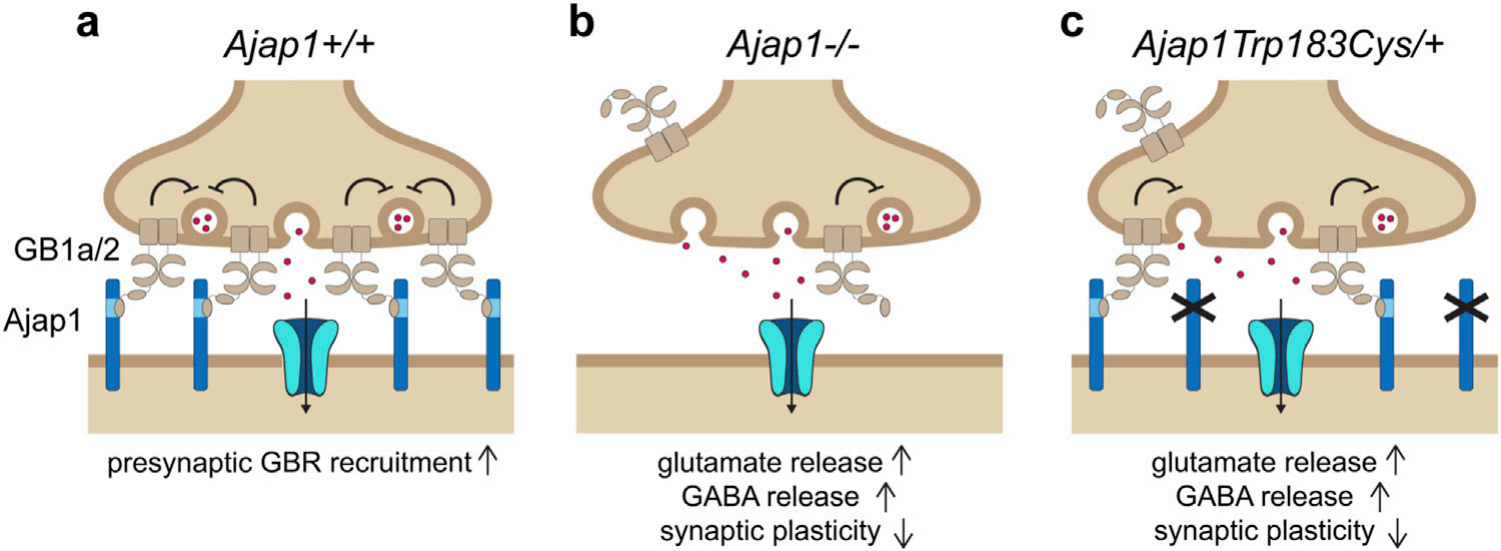
The pathogenic monoallelic *de novo AJAP1* p.Trp183Cys variant disrupts presynaptic GBR localization and function. **(a)** Under normal conditions, postsynaptic AJAP1 recruits GB1a/2 receptors to presynaptic terminals via a trans-synaptic interaction with the SD1 of the GB1a subunit. Presynaptic GB1a/2s receptors inhibit VGCCs (not shown), thereby regulating neurotransmitter release at both GABAergic and glutamatergic synapses. Ionotropic GABA or glutamate receptors are depicted in the postsynaptic membrane, along with inward currents (arrow). **(b)** In *Ajap1* ^−/−^ mice, the absence of AJAP1 impairs presynaptic GBR recruitment, leading to reduced inhibitory control over GABA and glutamate release, and resulting in deficits in synaptic plasticity. **(c)** The pathogenic monoallelic *de novo AJAP1* p.Trp183Cys variant, modeled in *Ajap* ^Trp183Cys/+^ mice, replicates the synaptic dysfunction observed in *Ajap1* ^*−/−*^ mice. This variant has a dysfunctional SD1 binding site, thereby impairing presynaptic localization of GBRs. As a result, GBR-mediated inhibition of neurotransmitter release is reduced, leading to deficits in synaptic plasticity and, in affected individuals, to seizures.

To strengthen a causal link between the p.Trp183Cys variant and GBR dysfunction, mice carrying the orthologous *Ajap1* p.Trp183Cys variant were generated. Heterozygous *Ajap1* ^Trp183Cys/+^ mice mimic the monoallelic p.Trp183Cys genotype observed in patients, enabling the investigation of GBR dysfunctions in the brain. Ultrastructural analysis revealed a significant reduction in presynaptic GBR levels in *Ajap1* ^Trp183Cys/+^ mice, demonstrating that replacement of tryptophan 183 impairs AJAP1’s ability to recruit GBRs to synaptic terminals (Figure 5). As a consequence, *Ajap1* ^Trp183Cys/+^ mice exhibited reduced GBR-mediated presynaptic inhibition at both excitatory and inhibitory synapses, along with impaired synaptic plasticity. Similar synaptic deficits were observed in *Ajap1* ^−/+^ mice, which model the heterozygous deletion of *AJAP1* seen in patients. Both *Ajap1* ^Trp183Cys/+^ and *Ajap1* ^−/+^ mice thus phenocopy the synaptic impairments reported in *GB1a* ^−/−^ mice, which lack presynaptic GBRs (Vigot et al., 2006). Individuals with heterozygous LOF alleles in *AJAP1* therefore represent the first clinical cases of presynaptic GBR dysfunction.

### 4.4 *PIANP* variants

PIANP is a single-pass transmembrane protein with sequence homology to AJAP1 (Dinamarca et al., 2019). Like AJAP1, it is a primary interaction partner of GBRs and binds to the SD1 domain of the GB1a subunit, albeit with a tenfold higher binding affinity (Dinamarca et al., 2019). The SD1-binding sites in PIANP and AJAP1 share only weak sequence homology, but PIANP also contains the conserved tryptophan residue essential for binding. Unlike AJAP1, PIANP lacks dendritic sorting motifs in its C-terminal intracellular domain and is expressed in both axons and dendrites. PIANP could therefore interact with GB1a in *cis* within axons and in *trans* across synapses in dendrites (Figure 1b). Interestingly, however, PIANP cannot compensate for the loss of AJAP1 at mossy fiber synapses (Fruh et al., 2024).

A case study described a boy with a homozygous nonsense variant in *PIANP*, who presented with global developmental delay (Winkler et al., 2020; Anazi et al., 2017) (Table 5). An additional individual with a heterozygous variant in PIANP was reported to exhibit musculoskeletal and nervous system abnormalities (Table 5). The synaptic effects of *PIANP* loss were investigated in *Pianp* knockout mice, which model the homozygous human condition. Electrophysiological recordings revealed a loss of presynaptic GBR-mediated inhibition at hippocampal synapses, suggesting that PIANP is required for stabilizing presynaptic GBRs, either in *cis* or through a trans-synaptic mechanism, similar to AJAP1 (Winkler et al., 2020). Behavioral phenotyping in mice demonstrated that *Pianp* deficiency leads to context-dependent increases in anxiety, spatial learning deficits, an altered stress response, severely impaired social interactions, and enhanced repetitive behaviors—all characteristic features of an autism spectrum disorder-like phenotype.

### 4.5 *KCTD8*, *KCTD12* and *KCTD16* variants

KCTD8, KCTD12, and KCTD16 interact with most GBRs in the brain and are considered auxiliary subunits of the receptor (Schwenk et al., 2010). They bind to both the receptor and the G protein, regulate the kinetics of the receptor response (Fritzius et al., 2024; Turecek et al., 2014), and also function as scaffolding proteins for effector channels such as VGCCs and HCN channels (Perez-Garci et al., 2025; Trovo et al., 2024). These roles suggest that dysfunctional KCTD proteins could contribute to pathologies associated with GBR dysfunction. GWAS have linked KCTD proteins to a range of neuropsychiatric and neurological conditions, including ASD, bipolar disorder, major depression, alcohol and opioid use disorders, insomnia, dementia, and brain development (Table 1). However, to date, no missense variants in *KCTD* genes have provided causal links to GBR-related pathologies.

## 5 Conclusion

Early pharmacological studies were instrumental in uncovering potential disease associations and suggesting therapeutic indications for GBR agonists and antagonists. Today, advances in genetic and genomic technologies enable the establishment of firm causal links between gene variants and human disease. As a widely adopted diagnostic tool, WES has facilitated the discovery of numerous missense variants in *GABBR1* and *GABBR2*—currently 80 in *GABBR1* and 433 in *GABBR2*, according to the ClinVar database at the time of this review. Missense and deletion variants have also been identified in *AJAP1* and *PIANP*, two proteins that selectively interact with presynaptic GBRs. Recombinant *in vitro* assays and mouse models have enabled the causal linking of several missense and deletion variants in *GABBR1*, *GABBR2*, *AJAP1*, and *PIANP* to a spectrum of neurodevelopmental disorders, including epileptic encephalopathy, Rett-like syndrome, global developmental delay, ID, ASD, and motor disorders. Among these, epilepsy is a frequent condition in individuals with *GABBR1* and *GABBR2* variants, consistent with the increased excitation–inhibition ratio and seizure susceptibility observed in GBR-deficient mice. While *GABBR1* and *GABBR2* variants affect both pre- and postsynaptic GBRs, *AJAP1* variants selectively impair presynaptic GBRs but result in clinical manifestations similar to LOF variants in *GABBR1* or *GABBR2*. In general, human phenotypes extend and refine insights gained from mouse models carrying equivalent variants. While such models are valuable for dissecting synaptic mechanisms, they have limited predictive power for complex neuropsychiatric and cognitive outcomes. Conversely, hyperalgesia—a robust phenotype in GBR-deficient mice—has not yet been causally linked to any known pathogenic variants in humans.

A large number of *GABBR1* and *GABBR2* VUS in ClinVar are found in individuals with phenotypes typically associated with GBR-related disorders, and many of these VUS receive high pathogenicity scores from *in silico* prediction tools. This suggests that a substantial proportion of currently unclassified variants may, in fact, be disease-causing. Functional validation in recombinant assay systems offers a rapid and cost-effective approach to assess the impact of such VUS on GBR function. These assays can discriminate between LOF and GOF effects, thereby facilitating the establishment of mechanistic links between receptor dysfunction and specific disease phenotypes. Notably, variants exhibiting similar properties in functional assay systems have been classified under distinct clinical diagnoses—for example, epileptic encephalopathy (EE) or Rett-like syndromes. This highlights the value of recombinant functional assays in enabling more accurate molecular diagnoses and refining genotype-phenotype correlations in affected individuals. A major bottleneck, however, is the limited availability of such functional platforms in clinical diagnostic settings—underscoring the need for scalable, robust assay systems and improved computational tools. Promising advances include the use of molecular dynamics simulations, which have been applied to predict constitutively active GBR states and to enhance conventional pathogenicity assessments. In addition, variants in GBR-associated proteins—such as Syt11, APP, and channels including VGCCs, HCN, and TRPV1—may contribute to GBR dysfunction and disease. However, as these proteins either modulate GBR trafficking or act as downstream effectors, their functional impact is challenging to assess using standard recombinant assay systems.

Accurate genetic diagnosis and a mechanistic understanding of disease pathology form the foundation for developing targeted, individualized treatment strategies. In the case of GBRs, a broad pharmacological toolkit is already available, including agonists, inverse agonists, and both positive and negative allosteric modulators. In principle, CRISPR/Cas technologies can be used to rapidly generate mouse models carrying specific, recurrent pathogenic variants—such as *GABBR2* p.Ala567Thr—providing a powerful platform for testing pharmacological interventions and advancing precision medicine approaches.
